# Generative artificial intelligence in medical education: from knowledge assessment to clinical reasoning and professional competence

**DOI:** 10.3389/fmed.2026.1855759

**Published:** 2026-08-25

**Authors:** Renxian Xie, Beien Zhang, Lifeng Xiao

**Affiliations:** 1 Department of Radiation Oncology, Cancer Hospital of Shantou University Medical College, Shantou, China; 2 Department of Science and Education, Cancer Hospital of Shantou University Medical College, Shantou, China; 3 Department of Emergency, Cancer Hospital of Shantou University Medical College, Shantou, China

**Keywords:** curriculum integration, generative artificial intelligence, knowledge assessment, large language models, medical education

## Abstract

Generative artificial intelligence (GenAI), particularly large language models (LLMs), is poised to fundamentally transform medical education. Based on a structured literature search of PubMed, Scopus, Web of Science, and Google Scholar, this review synthesizes current evidence on the applications, capabilities, and limitations of GenAI across the medical training continuum. Advanced LLMs demonstrate a formidable command of medical knowledge, consistently achieving passing scores on standardized licensing examinations, with GPT-4 and domain-specific models like Ortho GPT showing particular proficiency. As versatile teaching tools, these models can generate high-quality assessment materials, provide personalized on-demand tutoring, and power interactive virtual patients for clinical reasoning practice. However, this potential is tempered by significant challenges, including a propensity for “hallucinations,” embedded biases that can perpetuate health inequities, linguistic performance disparities, and a fundamental gap in flexible, adaptive clinical reasoning. Integration also raises critical concerns regarding academic integrity, potential over-reliance leading to deskilling, and data privacy. Responsible adoption requires a structured approach encompassing the development of tiered AI competency frameworks, blended curricular integration, dedicated faculty development, and a rigorous research agenda focused on longitudinal learning outcomes. Ultimately, GenAI should be viewed as a powerful augmentative tool, not a replacement for human educators. Its successful integration will depend on leveraging its strengths to enhance efficiency and scalability while preserving the essential humanistic elements of medical practice through expert oversight and validation.

## 1 Introduction

The advent of generative artificial intelligence (GenAI), particularly large language models (LLMs), represents a paradigm shift with profound implications for medical education. These models, capable of generating coherent text, interpreting images, and engaging in multi-turn dialogue, have rapidly transitioned from research novelties to tools with tangible applications across the learning continuum. The integration of AI into healthcare is no longer a distant prospect but an ongoing reality, compelling medical educators to understand, adapt, and harness these technologies to prepare future clinicians. This transformation is driven by the dual pressures of escalating demands on healthcare systems and the inherent limitations of traditional pedagogical methods, which often struggle with scalability, personalization, and the simulation of complex clinical reasoning.

Students and trainees are already at the forefront of this adoption, utilizing publicly available chatbots for tasks ranging from concept summarization and examination preparation to generating clinical vignettes and practicing diagnostic reasoning (1, 2). This grassroots integration occurs despite a significant gap in formal curricular guidance, raising urgent questions about efficacy, ethics, and the long-term impact on professional development. The potential of GenAI extends beyond mere information retrieval; it offers opportunities for creating dynamic, interactive learning environments, generating high-quality assessment materials, and providing personalized, on-demand tutoring. However, this promise is tempered by well-documented challenges, including the generation of plausible but incorrect information (hallucinations), embedded biases from training data, and concerns about the erosion of critical thinking and academic integrity. Several recent reviews have examined specific facets of generative AI in medical education, including its performance on licensing examinations (3), its utility in generating multiple-choice questions (4), and the design of large language model-based virtual patients (5, 6). While these contributions have advanced our understanding of isolated applications, the literature still lacks a comprehensive synthesis that spans the entire training continuum—from knowledge assessment and clinical reasoning to professional competence—and that offers an integrative conceptual framework to guide curriculum design. The present review addresses these gaps by critically examining the evidence across these interconnected domains and by proposing a unifying framework and competency model for responsible integration.

This review synthesizes the current evidence on the application of generative AI in medical education, drawing upon a burgeoning body of literature to map its capabilities, applications, and limitations. A structured search of the literature was performed in the following electronic databases: PubMed, Scopus, Web of Science, and Google Scholar. The search strategy combined terms related to generative AI (such as generative artificial intelligence, large language model, ChatGPT, GPT, AI chatbot, natural language processing) with terms related to medical education (such as medical education, clinical education, medical students, residency training, medical curriculum, undergraduate medical education, postgraduate medical education). The search was conducted from 2020 to February 2026, with a focus on publications from 2022 onward to reflect the rapid developments in this field. The reference lists of included articles and relevant reviews were also manually screened to identify additional sources. Articles were included if they directly addressed the application, evaluation, performance, or implications of generative AI models in medical or health professions education. Both original research studies and review articles were eligible. Editorials, commentaries, and non-English publications were excluded unless they provided substantive data or unique perspectives. Accordingly, this review examines the performance of GenAI in standardized knowledge assessment, evaluates its applications as a versatile teaching and learning tool, and investigates its role in clinical skills and reasoning training. It further discusses the significant implementation challenges and ethical considerations that must guide responsible use, and proposes future directions, including a tiered AI competency framework and strategies for curricular integration. The overall goal is to provide a comprehensive, evidence-informed overview that equips educators, institutions, and policymakers to navigate the opportunities and pitfalls of the AI-augmented future of medical education.

## 2 Performance of generative artificial intelligence models in standardized medical knowledge assessment

The systematic evaluation of large language models against medical knowledge benchmarks was catalysed by pivotal early studies that assessed ChatGPT's performance on the United States Medical Licensing Examination (USMLE). Kung et al. (7) and Gilson et al. (8) independently demonstrated that ChatGPT could achieve passing or near-passing scores, providing the initial proof-of-concept and igniting a global wave of subsequent benchmarking across specialties and languages. Since these foundational demonstrations, a primary area of investigation has been the systematic benchmarking of GenAI models against established standards of medical knowledge, using licensing and in-training examinations as proxies for clinical competence.

A primary area of investigation has been the benchmarking of GenAI models against established standards of medical knowledge, using licensing and in-training examinations as proxies for clinical competence. This body of work provides critical insights into the current capabilities and limitations of these models, revealing a landscape of impressive but uneven performance. Evaluations consistently show that advanced LLMs can meet or exceed the passing thresholds for major medical examinations, with performance often rivaling that of mid-level to senior trainees.

Studies across diverse specialties and geographic contexts confirm this trend. In orthopedics, the largest open-source vision-language models demonstrated performance comparable to second-year residents on the Orthopedic In-Training Examination (OITE), with model accuracy showing a steady increase with parameter size up to 72 billion (9). Similarly, in anesthesiology, LLMs were evaluated against Chinese residency examinations, with performance analysis extending to the reliability of clinical reasoning pathways (10). In Japan, LLMs were assessed using the National Medical Examination, with findings indicating “textbook-level” medical knowledge (11). A systematic review and meta-analysis of 36 studies concluded that the pooled accuracy of LLMs on medical licensing exams was 72%, with GPT-4 achieving the highest accuracy at 81% (3). This performance is not confined to English-language contexts; evaluations of the Chinese National Medical Licensing Examination (NMLE) found that models like DeepSeek-R1 significantly outperformed others like ChatGPT-4o, achieving high accuracy rates across multiple years (12). This pattern extends to specialized fields such as nephrology, where the newly released o1 pro model significantly outperformed GPT-4 on board renewal questions (13), and to radiation oncology, where GPT-5 demonstrated a mean accuracy of 92.8% on the ACR Radiation Oncology In-Training Examination, a substantial improvement over prior generations (14).

However, this strong overall performance masks significant variability and important caveats. Performance is highly dependent on model type, size, and specialization. Domain-specific models fine-tuned on medical literature, such as Ortho GPT, have been shown to outperform or match the strongest general-purpose models within their specialty, offering improved factual accuracy and contextual relevance (15). The “third wave” of GenAI models, featuring enhanced reasoning and research capabilities, has shown dramatic improvements, with more than half of tested models exceeding senior orthopedic resident performance on the OITE (16). Yet, significant disparities exist across subspecialties. For instance, in orthopedics, models consistently showed the most difficulty with spine and pediatric questions (9, 16). In occupational therapy, while models achieved passing-level scores on text-based questions, their performance markedly declined on visually based items (17).

The integration of multimodal capabilities—processing both text and images—is a frontier with mixed results. While some studies report high diagnostic accuracy for multimodal models on image-based challenges like the NEJM Image Challenge (18), others highlight persistent limitations. In orthopedic and histological image interpretation, even advanced models like GPT-4o demonstrated only moderate ability, with performance varying significantly by tissue type and magnification level (19). The reliability of citations and references generated by LLMs is another major concern. Studies have found rampant inaccuracies in provided citations, including fabricated publications and incorrect author, title, journal, and PMID details, even when responses themselves were moderately accurate (20). This underscores that high scores on knowledge assessments, while indicative of vast information recall, do not equate to reliable, citable expertise or nuanced clinical understanding. Therefore, while GenAI models have proven to be powerful tools for knowledge assessment and review, their outputs require rigorous expert validation, and their use in high-stakes evaluation settings must be approached with caution and clear understanding of their inherent limitations. Despite their impressive examination scores, the performance of GenAI models on standardized tests must be interpreted with caution, as high scores may not reflect genuine clinical competence. Standardized multiple-choice examinations primarily assess knowledge recall and pattern recognition, whereas real-world clinical practice demands flexible, adaptive reasoning, commonsense knowledge application, and the ability to navigate uncertainty and ambiguity. A critical study has documented a “multiple-choice illusion” in which large language models exhibited a 39.43% average performance decline when the same knowledge was tested via free-response questions—a drop substantially greater than that observed in human examinees (21). This finding suggests that MCQ benchmarks can significantly overestimate true clinical reasoning ability. Moreover, recent work utilizing medical abstraction and reasoning tasks has shown that current models struggle with cognitive flexibility, frequently failing to overcome the Einstellung effect and defaulting to rote pattern-matching rather than dynamic problem-solving (22). Such inflexible reasoning, combined with the well-documented propensity to hallucinate, highlights a fundamental gap between knowledge retrieval and the nuanced, context-dependent judgment that defines clinical practice. Consequently, strong examination performance should not be equated with clinical readiness; it merely demonstrates that models can exploit the statistical regularities in test items. Future evaluations of GenAI in medical education must move beyond multiple-choice benchmarks and incorporate free-response, simulation-based, and real-world clinical assessments to better capture the multifaceted nature of clinical competence. Table 1 summarizes the performance of various generative AI models on standardized medical examinations across different specialties, highlighting the variability in accuracy and the influence of model type, question format, and clinical domain.

**Table 1:** Performance of generative AI models on standardized medical examinations.

Specialty/examination	Models evaluated	Key findings
Orthopedic In-Training Examination (OITE) (9)	Open-source vision-language models	Performance comparable to second-year residents; accuracy increased with parameter size up to 72B
Chinese Anesthesiology Residency Examinations (10)	Multiple LLMs	Variable performance in clinical reasoning pathways; reliability concerns identified
Japanese National Medical Examination (11)	Various LLMs	Demonstrated “textbook-level” medical knowledge acquisition
Multiple licensing exams (Meta-analysis) (3)	36 studies, multiple models	Pooled accuracy: 72%; GPT-4 highest at 81%
Chinese National Medical Licensing Examination (12)	DeepSeek-R1, ChatGPT-4o	DeepSeek-R1 significantly outperformed ChatGPT-4o across multiple years
Nephrology Board Renewal (13)	o1 pro, GPT-4	o1 pro significantly outperformed GPT-4 on board renewal questions
ACR Radiation Oncology In-Training Examination (14)	GPT-5	Mean accuracy: 92.8%; substantial improvement over prior generations
Orthopedic Examination Questions (15)	Ortho GPT, general LLMs	Domain-specific model outperformed or matched general-purpose models
Occupational Therapy National Exam (17)	Multimodal LLMs	Passing scores on text; marked decline on visually-based items
Multiple-choice vs. free-response (21)	Various LLMs	39.43% performance decline in free-response vs. MCQ formats

LLM, large language model; OITE, Orthopedic In-Training Examination; GPT, Generative Pre-trained Transformer; ACR, American College of Radiology; MCQ, multiple-choice question; NBME, National Board of Medical Examiners; vs., versus.

## 3 The application of generative artificial intelligence as a diverse teaching and learning tool

Beyond assessment, generative AI is being actively explored and deployed as a dynamic tool to enhance both teaching methodologies and self-directed learning. Its applications are remarkably diverse, ranging from automated content creation and personalized tutoring to facilitating complex pedagogical strategies like case-based learning. This versatility addresses several long-standing challenges in medical education, including resource intensity, lack of personalization, and the need for scalable, engaging learning materials.

One of the most prominent applications is the generation of educational content. LLMs can rapidly produce high-quality, customizable teaching materials, such as multiple-choice questions (MCQs), clinical vignettes, and structured explanations. Systematic reviews indicate that, with expert oversight, models like ChatGPT-4 can generate MCQs of similar quality to human-authored items in terms of relevance, clarity, and distractor quality (4). This capability has been successfully piloted in specific disciplines, such as generating clinically integrated pathology questions directly from lecture slides (23) and creating USMLE-style practice questions aligned with NBME guidelines (24). The efficiency gains are substantial, offering a solution to the time-consuming and inconsistent traditional methods of case and question creation (25). Furthermore, AI can generate visual educational aids, such as anterior segment images in ophthalmology, which, while most effective for pathologies with distinct morphological features, offer a scalable resource for early-stage trainees (26).

As a personalized learning assistant, GenAI provides 24/7, on-demand support that complements traditional instruction. Students widely report using chatbots like ChatGPT for concise topic summarization, clarification of complex concepts, and generation of practice questions (27). Structured implementations, such as a DeepSeek-based teaching assistant in anesthesiology theory courses, have shown significant improvements in short-term knowledge acquisition and learner engagement compared to traditional teaching alone (28). These tools can adapt to individual learning paces and needs, offering a form of interactive tutoring that was previously difficult to scale. For instance, an AI-driven platform providing personalized feedback on exam performance (“Sisu Athwala”) was well-received by students and validated by expert mentors, demonstrating potential to augment human mentoring where resources are constrained (29).

GenAI also enables innovative pedagogical models. It can act as a “librarian,” guiding students in preparing for context-specific learning, such as intraoperative learning during surgical clerkships, by helping them dictate learning objectives and receive directed resources (30). It facilitates case-based learning (CBL) by generating diverse, nuanced patient cases and simulating patient responses, allowing learners to practice diagnostic reasoning in a low-risk environment (31). In research education, a mentor-guided framework using AI for narrative review writing improved students’ research readiness, literature search skills, and understanding of ethical AI use (32). Additionally, AI can streamline administrative and orientation tasks; a custom-trained chatbot significantly improved international medical students’ ability to locate and understand key course logistics compared to a standard e-learning platform (33).

The effectiveness of these tools is influenced by factors explored in technology acceptance studies. For medical students, perceived usefulness and ease of use are strong direct predictors of their intention to use AI educational agents, while social influence appears less impactful (34). The concept of task-technology fit (TTF)—the alignment between the AI's capabilities and the learning task—is positively associated with both perceived usefulness and responsible use behavior (35). Importantly, the role of human educators evolves but remains central. Teacher support, encompassing affective, capacity, and behavioral dimensions, directly and indirectly enhances students’ academic self-efficacy and learning outcomes when using GenAI tools (36). Students consistently emphasize that AI is a complementary tool, not a replacement, and that expert validation and guidance are essential to ensure accurate learning and mitigate risks like over-reliance (37). Thus, the most promising educational applications of GenAI are those that leverage its power for content generation and personalized interaction within a structured, pedagogically sound framework overseen by skilled educators. Table 2 provides a comprehensive overview of generative AI applications as teaching and learning tools in medical education, categorizing them by purpose, implementation examples, educational benefits, and associated limitations.

**Table 2:** Applications of generative AI as teaching and learning tools in medical education.

Application category	Representative use	Key benefits	Key limitations
Content Generation	MCQ and vignette creation; visual educational aids (4, 23, 24, 26)	Efficient, scalable, customizable	Requires expert quality control; may lack clinical nuance
Personalized Learning & Support	AI teaching assistants; automated feedback; chatbot-based orientation (28, 29, 33)	24/7 access; improved engagement and short-term knowledge; efficient logistics	Risk of over-reliance; needs curricular alignment
Innovative Pedagogy	Case-based learning with simulated responses; AI-assisted research writing (31, 32)	Low-risk reasoning practice; enhanced research readiness	Variable realism; requires structured faculty guidance
Clinical Skills & Reasoning	Virtual patient history-taking; AI-generated Script Concordance Tests; team-based simulation (ACLS) (5, 39, 40)	Standardized, repeatable practice; automated reasoning assessment	Dialogue authenticity limited; requires expert validation; still in pilot stages

AI, artificial intelligence; MCQ, multiple-choice question; ACLS, Advanced Cardiovascular Life Support.

## 4 Integration of generative artificial intelligence in clinical skills and reasoning training

The simulation of clinical environments and the development of diagnostic reasoning represent a particularly promising and complex domain for GenAI integration. By powering virtual patients (VPs) and interactive simulations, AI offers opportunities for repetitive, standardized practice of history-taking, communication, and clinical decision-making—skills traditionally honed through resource-intensive encounters with standardized patients or in clinical rotations.

LLM-based virtual patient systems are emerging as scalable tools for history-taking practice. A comprehensive systematic review identified 39 studies in this area, noting that these systems primarily simulate internal medicine and mental health disorders, often using techniques like role-based prompts and knowledge graph integration to enhance dialogue realism and diagnostic accuracy (5). The educational promise is significant; such systems allow learners to practice formulating questions, interpreting responses, and building differential diagnoses in a safe, feedback-rich environment. Comparative studies suggest that more advanced implementations, such as AI-enhanced social robotic VPs, can offer superior design characteristics—including greater authenticity and professional approach—compared to conventional computer-based VP platforms, leading to stronger student preference for their use in clinical reasoning training (38).

GenAI is also being leveraged to teach and assess the nuanced process of clinical reasoning itself. Tools have been developed to generate and evaluate Script Concordance Tests (SCTs), which measure reasoning in conditions of uncertainty. In a formative evaluation, trained LLMs were able to reliably simulate expert judgment in generating SCT scoring keys and feedback for cardiology and pneumology, with the AI-generated feedback being rated as helpful by students (39). This points to a potential avenue for automating aspects of reasoning assessment while maintaining pedagogical value. Furthermore, AI-driven simulations are being used for team-based skills training. A pilot study developed an LLM-based simulation platform with AI agents acting as team members in Advanced Cardiovascular Life Support (ACLS) scenarios, which consistently adhered to clinical guidelines and showed potential as an adjunct for team leadership training (40). Similarly, for psychological first aid (PFA) training, an LLM chatbot effectively simulated individuals in post-stress scenarios, enabling trainees to practice therapeutic conversations flexibly and repeatedly (41).

However, integrating GenAI into clinical skills training reveals profound challenges related to the authenticity and flexibility of AI reasoning. While models excel at pattern recognition on structured tasks, they often struggle with the flexible, adaptive reasoning required in real-world clinical problem-solving. Studies designed to probe these limitations, such as those using a Medical Abstraction and Reasoning Corpus (mARC-QA), find that LLMs perform poorly compared to physicians on scenarios requiring them to overcome cognitive fixation (the Einstellung effect) and apply commonsense medical knowledge (22). The models tend to exhibit inflexible reasoning, a propensity to hallucinate, and overconfidence in incorrect answers. This underscores a critical gap between performance on knowledge tests and true clinical reasoning capability.

These limitations are not incidental but arise from fundamental architectural and training characteristics of current LLMs. First, these models lack genuine causal reasoning; they operate as statistical pattern-matching engines trained to predict subsequent tokens, without explicit knowledge of the underlying causal mechanisms in pathophysiology or pharmacology (42). Consequently, they may identify associations but fail to construct the coherent causal chains required for diagnostic inference. Second, despite advances in context-window length, LLMs exhibit limited contextual understanding: they struggle to integrate subtle, multimodal clinical cues—such as facial expressions, tone of voice, and the temporal evolution of symptoms—that experienced clinicians naturally synthesize to refine their assessments (22). Third, LLMs are devoid of experiential learning; unlike clinicians whose knowledge is continuously updated through direct patient encounters, outcomes, and reflective practice, these models rely on static pre-training datasets and cannot learn from individual cases or feedback in real time (42). This absence of embodied experience prevents the development of pattern-recognition heuristics that are honed over years of clinical practice. Finally, LLMs face profound challenges in managing uncertainty and ambiguity, which are intrinsic to medical decision-making. Rather than quantifying or calibrating uncertainty, models tend to default to confident assertions even when presented with incomplete or equivocal data, generating overconfident and potentially erroneous recommendations. These intertwined deficits explain why strong performance on structured knowledge tests does not translate into flexible, context-sensitive clinical reasoning, and they reinforce the necessity of cautious, hybrid integration strategies that combine AI capabilities with expert human oversight.

The role of prompt engineering and interaction design becomes paramount in this context. Effective use of techniques like chain-of-thought prompting and few-shot learning can significantly improve model outputs and partially correct initial reasoning errors (18, 43). This shifts the interaction from a simple informational exchange to an active learning experience, where the learner must provide clear context and learning goals to guide the AI (44). The concept of “productive struggle”—deliberate engagement with complex issues—is essential, and educators must design interactions that foster this rather than allowing passive receipt of AI-generated answers (45). Ultimately, while GenAI offers unprecedented tools for simulating clinical encounters and practicing reasoning frameworks, its current limitations necessitate a hybrid model. The most effective training will likely combine AI-powered simulations for accessibility and repetition with expert-facilitated debriefing, validation of reasoning pathways, and immersion in real clinical environments to develop the adaptive, humanistic judgment that remains the cornerstone of medical practice.

## 5 Implementation challenges, ethical considerations, and responsible use

The integration of generative AI into medical education is fraught with significant practical, ethical, and societal challenges that must be proactively addressed to ensure safe, equitable, and effective implementation. These concerns extend beyond technical accuracy to encompass issues of bias, academic integrity, professional deskilling, data privacy, and the evolving social dynamics within healthcare.

A foremost ethical concern is the pervasive risk of bias and health inequity. LLMs trained on historical data can perpetuate and even amplify existing disparities. This has been empirically demonstrated; for instance, GPT-4o underrepresented women in simulated cardiovascular cases and diagnosed female patients with key conditions like coronary artery disease less accurately than males, reinforcing historical gender biases in medicine (46). Linguistic disparities are another critical issue, with studies showing that leading LLMs provide clinically less reliable information in languages like Spanish compared to English, potentially exacerbating health inequities for non-English speaking populations (47). These biases are embedded in the training data and model architecture, requiring deliberate efforts in bias detection, mitigation, and the use of diverse, representative datasets for model training and fine-tuning (48).

The reliability of AI-generated content, particularly the phenomenon of “hallucination” or fabrication, poses a direct threat to learning integrity. Models can generate confident, coherent, but entirely incorrect information and citations, which inexperienced learners may struggle to detect (20). This risk necessitates the development of critical AI literacy among students, teaching them to verify outputs against trusted sources and understand the probabilistic nature of LLMs. Furthermore, the ease of access to AI tools raises profound questions about academic integrity, particularly in assessment. The traditional personal statement for admissions, for example, may be undermined by AI generation, prompting calls to refocus the process on reflection and human growth rather than polished narrative output (49). Similarly, the potential for surreptitious use of AI in virtual interviews for admissions, while shown in one experiment not to confer a performance advantage, remains a concern that requires procedural safeguards (50).

Widespread adoption risks fostering over-reliance and potential deskilling. Students and interns report concerns that defaulting to AI for answers could erode their own critical thinking abilities and clinical judgment (51). The “black box” nature of complex models, where the reasoning process is not transparent, complicates this further, as learners cannot fully interrogate the logic behind an AI's suggestion (42, 48). This underscores the irreplaceable role of the educator as an expert validator and guide who can contextualize AI outputs, highlight reasoning gaps, and ensure that foundational knowledge and cognitive skills are firmly developed in the learner (37).

Implementation is also hampered by practical barriers: uneven digital infrastructure, variable digital literacy among faculty and students, high costs for advanced models, and concerns about data privacy—especially when using patient data or student interactions to train models (48, 52, 53). Perhaps most subtly, the integration of AI may reshape professional hierarchies and collaboration. A thematic analysis of online discourse among pre-meds, medical students, and physicians revealed a socially hierarchical perception of which medical roles AI would replace first, with “lower” status roles seen as most vulnerable. This biased mindset could contribute to role devaluation and mistrust within healthcare teams (54). Therefore, responsible use requires a multi-layered approach: robust technical safeguards, clear ethical guidelines and governance frameworks, comprehensive education in AI literacy for both students and faculty, and an ongoing commitment to equity, transparency, and the preservation of humanistic values at the core of medicine. Several limitations of this narrative mini-review should be noted. First, this is not a systematic review; although a structured literature search was performed, formal quality or risk-of-bias appraisal was not conducted, which limits full reproducibility. Second, the field of generative AI is evolving rapidly, and specific performance figures for individual model versions may quickly become outdated; however, the thematic trends and the proposed conceptual and competency frameworks are expected to remain relevant. Third, the reviewed studies are heterogeneous in design, educational setting, model, and outcome measure, precluding direct quantitative comparisons. Fourth, the existing evidence is predominantly cross-sectional or short-term, and the longitudinal impact of GenAI on knowledge retention and professional development remains largely unexplored. Fifth, only English-language publications were systematically included, which may introduce cultural and linguistic bias. Finally, much of the evidence relies on standardized examination performance as a proxy for clinical competence—a metric that this review itself critically examines. These limitations should be considered when interpreting the findings and when designing future longitudinal and multi-language studies.

## 6 Future directions: competency frameworks, curriculum integration, and research agenda

**Figure 1:**
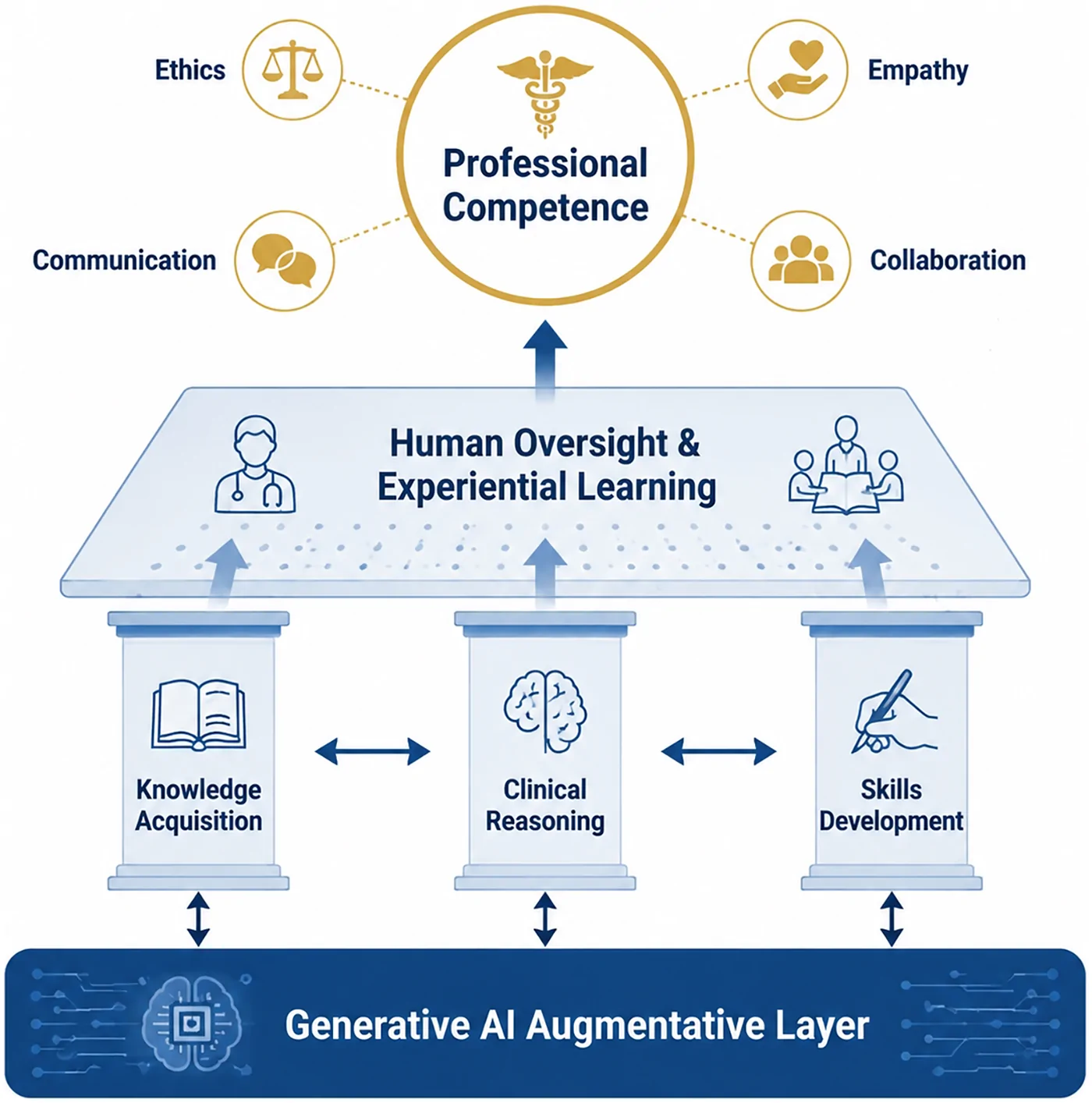
Conceptual framework illustrating the integration of generative artificial intelligence (GenAI) across the domains of medical education and professional development.

**Figure 2:**
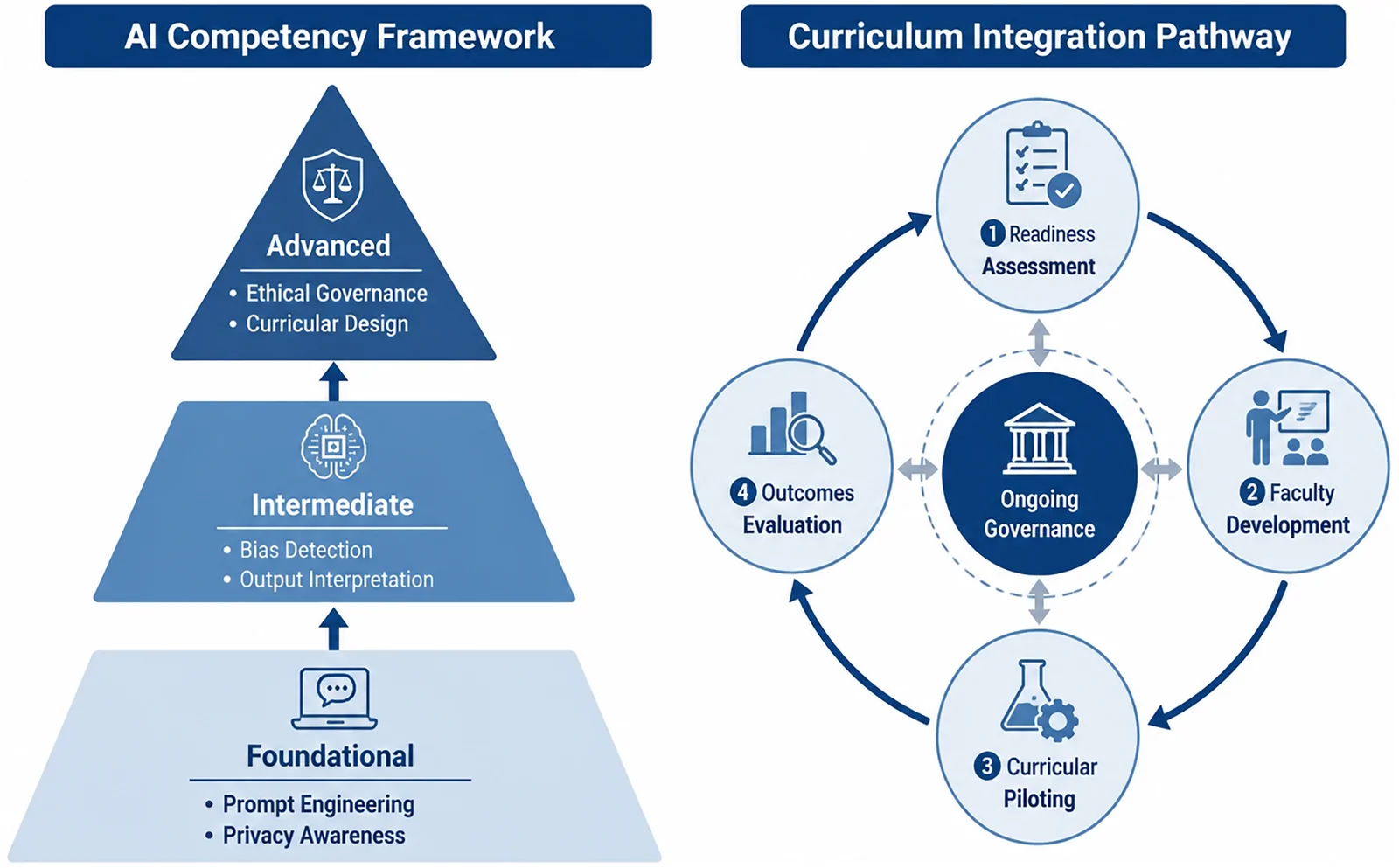
Tiered AI competency framework and pathway for responsible curriculum integration.

The conceptual framework (Figure 1) and the tiered competency and curriculum-integration framework (Figure 2) were derived inductively from the literature reviewed and refined through iterative author consensus. They are presented as proposed conceptual models that require future empirical validation and have not undergone formal external review.

Building on the evidence reviewed, we propose a unifying conceptual framework that synthesizes the core domains of GenAI integration in medical education (Figure 1). In this model, GenAI functions as an augmentative layer that interfaces bidirectionally with three foundational pillars of professional development: knowledge acquisition, clinical reasoning, and procedural skills development. This bidirectional interface means that GenAI tools not only deliver content and simulations but also capture learner responses, enabling adaptive feedback and iterative improvement of instructional strategies under educator supervision. GenAI tools can accelerate factual learning, generate assessment materials, and power virtual patient simulations, yet their outputs require continuous critical evaluation. Crucially, the pathways connecting knowledge, reasoning, and skills are mediated by human oversight and experiential learning, which remain indispensable for cultivating the flexible, context-sensitive judgment that characterizes clinical expertise. At the apex of this framework lies professional competence—encompassing ethical decision-making, empathy, communication, and collaborative practice—which is refined through mentored clinical experiences and cannot be supplanted by AI. This perspective emphasizes that GenAI should be embedded within an educational ecosystem where technology enhances, rather than replaces, the long-term, humanistic processes of becoming a clinician.

As generative AI becomes an indelible part of the medical landscape, a proactive and structured approach is required to guide its integration into education. Future efforts must focus on developing standardized competency frameworks, designing effective curricular pathways, and pursuing a rigorous research agenda that moves beyond descriptive studies to measure educational impact and long-term outcomes.

A critical priority is the definition of AI-specific competencies for medical learners and clinicians. Proposals are emerging for tiered frameworks that outline the skills required for safe and effective use. One such framework suggests three levels: foundational skills (prompt engineering, privacy awareness), intermediate skills (bias detection, interpreting AI outputs), and advanced leadership skills (ethical governance, model lifecycle management) (55). These competencies must be integrated into existing milestone frameworks across the continuum of undergraduate, postgraduate, and continuing medical education. For example, foundational AI literacy can be introduced through early undergraduate coursework, intermediate skills applied during clinical placements with AI-assisted decision tools, and advanced governance competencies cultivated through faculty development and leadership programs. A schematic overview of the proposed competency framework and the institutional pathway for responsible GenAI integration is presented in Figure 2. The three-tier competency model (foundational, intermediate, and advanced) maps the progression from basic AI literacy to leadership in ethical governance, while the integration pathway outlines a cyclical institutional process: conducting readiness assessments, developing faculty capacity, piloting curricular initiatives, evaluating outcomes, and scaling successful practices under continuous oversight. This visual synthesis offers educators and policymakers a scaffolded, actionable blueprint for embedding GenAI into medical training in a structured and ethically grounded manner. Parallel to learner competencies, dedicated faculty development programs are urgently needed to equip educators with the knowledge and skills to teach with and about AI, to critically evaluate AI-generated content, and to redesign assessments for an AI-augmented world (56).

Curricular integration strategies should move beyond isolated tool demonstrations toward a blended, scaffolded approach. AI literacy should be introduced early, framing AI as a tool to be mastered critically. This can progress to using AI for specific pedagogical purposes, such as generating differential diagnoses, practicing patient communication via virtual patients, or receiving personalized feedback on clinical notes (45, 57). The “librarian” model, where AI guides personalized preparation for clinical experiences, represents another promising integrative approach (30). Crucially, curricula must also address the limitations of AI, fostering discussions on ethics, bias, and the irreplaceable elements of human judgment and compassion. Assessment methods themselves must evolve; over-reliance on multiple-choice questions, which can overestimate AI (and potentially human) capability, should be balanced with more robust evaluations of clinical reasoning, such as AI-evaluated free-response questions or observed structured clinical examinations that test the integration of AI-assisted information into patient care decisions (21).

The research agenda must shift from benchmarking studies to more nuanced investigations of learning processes and outcomes. Key areas include: longitudinal studies on the impact of AI tool use on knowledge retention, critical thinking, and clinical performance; comparative effectiveness research of different AI-integrated pedagogical models; development and validation of standardized metrics for evaluating AI-generated educational content and virtual patient interactions (5, 6); and exploration of human-AI collaboration dynamics in clinical reasoning tasks (22). Research must also address equity, exploring how to mitigate biases in AI tools and ensure they reduce rather than widen educational and health disparities (47). Implementation science research is needed to identify best practices for institutional adoption, including governance models, infrastructure requirements, and strategies to foster a culture of responsible innovation.

Finally, interdisciplinary collaboration and international consensus-building are essential. Educators, clinicians, data scientists, ethicists, and learners must co-create guidelines and standards. The rapid evolution of the technology demands an agile, evidence-informed approach to curriculum development and policy, ensuring that the medical professionals of the future are not only proficient users of AI but also wise stewards who can harness its power to enhance patient care while upholding the fundamental values of the medical profession.

## 7 Conclusion

Generative artificial intelligence is poised to substantially transform medical education, offering transformative opportunities alongside formidable challenges. The evidence synthesized in this review demonstrates that advanced LLMs possess a formidable command of medical knowledge, capable of performing at or above the level of trainees on standardized examinations across numerous specialties and languages. This capability underpins a wide array of applications, from automating the generation of assessment materials and personalized tutoring to powering interactive virtual patients for clinical reasoning practice. However, the current evidence base is predominantly cross-sectional and focused on short-term knowledge acquisition; robust longitudinal data on clinical performance, professional development, and patient outcomes remain limited. Moreover, the potential of GenAI is tightly constrained by significant limitations, including hallucinations, embedded biases, linguistic disparities, and a fundamental gap in flexible, commonsense clinical reasoning, revealing that current models are sophisticated pattern-matching tools rather than autonomous experts.

These findings lead to several recommendations. The path forward requires a deliberate, critical, and human-centric approach. Drawing on the reviewed evidence and our proposed conceptual and competency frameworks, medical educators and institutions should develop structured AI competency frameworks, redesign curricula to foster AI literacy alongside robust clinical reasoning, and implement faculty development programs. Research must advance beyond performance benchmarks to investigate longitudinal learning outcomes and the optimal design of human-AI collaborative learning. Ethical principles of equity, transparency, and accountability must be woven into every stage of integration.

Ultimately, generative AI should be viewed as a powerful augmentative tool, not a replacement for human educators. Its greatest value may lie in handling repetitive tasks and providing scalable practice environments, thereby freeing human expertise for higher-order teaching—mentoring, nuanced feedback, and modeling the humanistic aspects of care. By embracing this complementary role with an awareness of its limits, the medical education community can harness AI to develop future physicians who are knowledgeable, critically thoughtful, and ethically grounded. The goal remains to train students to lead AI, ensuring that technology enhances rather than diminishes the art and science of medicine.
